# Copper-Induced Activation of MAPKs, CDPKs and CaMKs Triggers Activation of Hexokinase and Inhibition of Pyruvate Kinase Leading to Increased Synthesis of ASC, GSH and NADPH in *Ulva compressa*

**DOI:** 10.3389/fpls.2020.00990

**Published:** 2020-07-09

**Authors:** Daniel Laporte, Alberto González, Alejandra Moenne

**Affiliations:** Laboratory of Marine Biotechnology, Faculty of Chemistry and Biology, University of Santiago of Chile, Santiago, Chile

**Keywords:** ascorbate, CaMKs, CDPKs, copper, glutathione, MAPKs, NADPH, *Ulva compressa*

## Abstract

In order to analyze whether copper induces activation of CaMK, CDPK and/or MAPK signaling pathways leading to carbon flux reprogramming and to the synthesis of ascorbate (ASC), glutathione (GSH) and NADPH in order to buffer copper-induced oxidative stress, *U. compressa* was initially cultivated with 10 µM copper for 0 to 10 days. The activities of hexokinase (HK), pyruvate kinase (PK), L-galactone 1,4 lactone dehydrogenase (L-GLDH) and glucose 6-P dehydrogenase (G6PDH) were analyzed. HK activity was increased whereas PK was inhibited, and L-GLDH and G6PDH activities were increased indicating a copper-induced modulation of glycolysis leading to carbon flux reprogramming. Then, the alga was cultivated with an inhibitor of CaMs and CaMKs, CDPKs and MAPKs, and with 10 µM of copper for 5 days and the activities of HK, PK, L-GLDH, G6PDH and glutathione synthase (GS), the levels of ASC/DHA, GSG/GSSG and NADPH/NADP, the levels of superoxide anions (SA) and hydrogen peroxide (HP) and the integrity of plasma membrane were determined. The activation of HK was dependent on MAPKs, the inhibition of PK on CDPKs/MAPKs, the activation of L-GLDH on MAPKs, the activation GS on CDPKs/MAPKs, and the activation of G6PDH on MAPKs. Increases in the level of ASC/DHA were dependent on activation of CaMKs/CDPKs/MAPKs, those of GSG/GSSG on MAPKs and those NADPH/NADP on CaMKs/CDPKs/MAPKs. The accumulation of superoxide anions and hydrogen peroxide and the integrity of plasma membrane were dependent on CaMKs/CDPKs/MAPKs. Thus, copper induced the activation of MAPKs, CDPKs and CaMKs leading to the modulation of glycolysis and carbon flux reprogramming which trigger an increase in ASC, GSH and NADPH syntheses and the activation of antioxidant enzymes in order to buffer copper-induced oxidative stress in *U. compressa*.

## Introduction

Essential heavy metals are those required for proper function of proteins and enzymes and they are iron, copper, zinc, vanadium and nickel in animals and plants (Yadav, 2010). In addition, cadmium is an essential heavy metal for marine phytoplankton since it is required for the function of carbonic anhydrase (Xu and Morel, 2013). In particular, copper is an essential cofactor of several proteins and enzymes such as platocyanin, citochrome c oxidase, laccase, Cu/Zn superoxide dismutase and several other oxidases and dehydrogenases (Yadav, 2010; Ali et al., 2019). However, copper in excess induces oxidative stress due to the accumulation of reactive oxygen species (ROS) that can oxidize biological macromolecules such as lipids, proteins, nucleic acids and sugars resulting harmful for cells (Yadav, 2010; Ali et al., 2019). Some heavy metals are not required for the function of proteins and enzymes and they are designated as non-essential heavy metals such as lead, arsenic, mercury, silver and others.

Essential and non-essential heavy metals can induce oxidative stress due to the synthesis of ROS such as superoxide anions and hydrogen peroxide in plants (Yadav, 2010; Ali et al., 2019). The increase in ROS can be mitigated by the activation of antioxidant enzymes such as superoxide dismutase (SOD), that dismutate superoxide anions to hydrogen peroxide, and catalase (CAT), ascorbate peroxidase (AP), glutathione peroxidase (GP) and peroxiredoxin (PRX) that reduce hydrogen peroxide. In addition, the increase in ROS induces the activation of enzymes that synthesize antioxidant molecules ASC, GSH and NADPH, thus, increasing the level of substrates used by AP, DHAR, GP and glutathione reductase (GR) (Foyer and Noctor, 2011). Important antioxidant enzymes are those that constitute the Haliwell–Asada–Foyer (HAF) cyle that operates in chloroplasts: AP, that oxidizes ASC to dehydroascorbate (DHA); dehydroascorbate reductase (DHAR), that reduces DHA to ASC by oxidizing GSH to oxidized glutathione (GSSG); and GR, that reduced GSSG to GSH using NADPH as reducing power (Foyer and Noctor, 2011).

ASC can also be directly oxidized by ROS such as superoxide anions and hydrogen peroxide producing dehydroascorbate (DHA) (Foyer and Noctor, 2011). In addition, ASC is also a cofactor of de-epoxidases in the xanthophyll cycle that protects thylakoid membranes from lipid oxidation (Smirnoff, 2011; Wheeler et al., 2015). Moreover, ASC prevent photoinhibition by donating electrons to electron transport chains in plants exposed to high light (Smirnoff, 2011; Wheeler et al., 2015). Thus, ASC has a triple function in plants acting as the substrate of AP and de-epoxidases, and as a direct electron donor. In plants, ASC is synthesized from glucose-6P (G6P) and the two last steps of ASC synthesis are displayed by L-galactose dehydrogenase (L-GDH) and L-galactono 1,4 lactone dehydrogenase (L-GLDH) (Wheeler et al., 1998; Valpuesta and Botella, 2004). In animals, ASC is also synthesized from G6P but the two last steps are performed by aldono lactonase and guluno-lactone dehydrogenase (GuLDH) (Wheeler et al., 1998; Valpuesta and Botella, 2004). Interestingly, most animals can synthesize ASC in the liver but humans and primates present mutations in the gene encoding GuLDH (Nishikimi and Unfriend, 1976; Nishikimi et al., 1994) and they need to acquire ASC from the diet.

GSH can also be directly oxidized by ROS producing GSSG (Foyer and Noctor, 2011; Lu, 2013). GSH is constituted by three amino acids, glutamate, cysteine and glycine and it is synthesized by the enzymes *γ*-glutamyl cysteinyl synthase (GCS) and glutathione synthase (GS), and both enzymes require ATP (Foyer and Noctor, 2011; Lu, 2013). Glutamate is formed by the amination of α-ketoglutarate produced in the Krebs cycle and it is the precursor of other amino acids such as glutamine, aspartate, proline, arginine and asparagine, and the neurotransmitter *γ*-aminobutyric acid (GABA) (Forde and Lea, 2007). Serine is synthesized from 3-phophoglycerate produced in glycolysis and it is the precursor of methionine, cysteine and glycine (Ros et al., 2014). In addition, GSH is the precursor of phytochelatins (PCs) that are formed by condensation of GSH units by the enzyme phytochelatin synthase (Cobbet and Goldsbrough, 2002; Yadav, 2010). PCs can also directly act as antioxidant molecules but their principal function is to sequester heavy metals ions, thus, reducing oxidative stress (Tsuji et al., 2002; Yadav, 2010). NAD and NADP are synthesized from aspartate and this amino acid is obtained from glutamate in plants (Noctor et al., 2006). In addition, NADPH is synthesized by the reduction of NADP by ferredoxin NADP-reductase in chloroplats and/or by the enzyme glucose 6-P dehydrogenase (G6PDH) in the cytosol. NADPH is the final electron donor in antioxidant systems since it is the substrate of GR, the last enzyme of HAF cycle, and the substrate of NADPH-thioredoxin reductase that reduced thioredoxins that, in turn, reduced PRX that consumes hydrogen peroxide (Valderrama et al., 2006).

The marine macroalga *Ulva compressa* (Chlorophyta) is the dominant species in copper and heavy metal-contaminated coastal areas in northern Chile and all over the world (Villares et al., 2002; Ratkevicius et al., 2003). This marine macroalga has been extensively studied in regard to the mechanisms involved in copper tolerance (González et al., 2010; González et al., 2012; Mellado et al., 2012; Laporte et al., 2016; Moenne et al., 2016) and copper accumulation (Ratkevicius et al., 2003; Navarrete et al., 2019; Zúñiga et al., 2020). It has been shown that this alga presents exceptional mechanisms of copper tolerance that will be mentioned bellow (Laporte et al., 2020; Moenne et al., 2020). It was initially shown that the alga collected in copper-contaminated sites displayed and increase in intracellular copper level, AP activity and synthesis of ASC, which was accumultated as DHA (Ratkevicius et al., 2003). The alga cultivated *in vitro* with a sub-lethal concentration of copper (10 µM) for 7 days showed an increase in activities of antioxidant enzymes such as SOD, AP and GR (González et al., 2010; González et al., 2012). In addition, copper induced an increase in the level of antioxidant molecules ASC, GSH and PCs until day 7 (Mellado et al., 2012). Interestingly, an increase ASC is due to an increase in GDH and L-GLDH activities, whereas no GulDH activity was detected (Mellado et al., 2012). In addition, the activities of GCS and GS involved in GSH synthesis were also increased until day 7 (Mellado et al., 2012). On the other hand, transcriptomic analyses were performed using Illumina technique and total RNA of the alga cultivated with 10 µM for 0 to 24 h (Rodríguez et al., 2018) and for 0 to 5 days (Laporte et al., 2020). Differential expressed transcripts showed an increase in the level of transcripts encoding several subunits of photosystem II (PSII) and PSI as well as proteins and enzymes involved in repair of PSII, protection of PSI suggesting that net photosynthesis may be increased. In fact, there was an increase in net photosynthesis that may lead to an increase NADPH levels (Rodríguez et al., 2018; Laporte et al., 2020). In addition, there was an increase in activities of rubisco, involved in C assimilation, glutamine synthase, involved in N assimilation, and O-acetyl thiol lyase, involved in S assimilation, which are reductive processes that require NADPH. Thus, photosynthesis and C, N and S assimilation are increased in the alga exposed to copper stress (Laporte et al., 2020).

In this work, we analyzed whether copper may activate CaMKs, CDPKs and/or MAPKs signaling pathways which may regulate glycolysis inducing a carbon flux reprogramming that may increase the synthesis of ASC, GSH and NADPH in order to buffer copper-induced oxidative stress and membrane damage.

## Materials and Methods

### Algal and Seawater Sampling

The alga *U. compressa* was collected in Cachagua, a non-contaminated site located in central Chile (32° 34´S), transported to the laboratory in a cooler with ice. The alga was rinsed once in seawater, cleaned manually and sonicated twice for 3 min in an ultrasound bath (Hilab Innovation Systems). Seawater was obtained in Quintay (33° 12´S), another non-contaminated site of central Chile, filtered and kept at 4°C.

### *In Vitro* Cultures and Treatment With Inhibitors

The alga (1 g of fresh tissue) was cultivated in 3 ml of seawater without copper addition (control) or with 10 µM CuCl_2_ (Merck, Germany) for 0, 1, 3, 5, 7 and 10 days, in triplicate. The control and copper-supplemented culture medium was changed every 48 h. In addition, the alga (1 g FT) was cultivated in 3 ml of seawater without copper addition (control), with 10 µM of copper, or with 100 µM W-7, an inhibitor of calmodulins (CaMs) and CaM-dependent kinases (CaMKs), 10 µM of staurosporine (St), an inhibitor of calcium-dependent protein kinases (CDPKs), and 5 µM of PD-98059, an inhibitor of the MAPKK MEK1/2, and with 10 µM of copper for 5 days, in triplicate. It is important to mention that these inhibitors did not induced membrane damage or cell death in the alga cultivated without or with copper for 5 days. The control medium containing the inhibitor and copper was changed every 48 h.

### Preparation of Protein Extracts

The alga (1 g of FT) was frozen with liquid nitrogen and homogenized in a mortar using a pestle. Three milliliters of 100 mM phosphate buffer (pH 7.0) supplemented with 5 mM β-mercaptoethanol were added and the homogenization of the mixture was pursued until thawing. The mixture was centifuged at 14,000 rpm for 15 min at 4°C and the supernatant was recovered. Proteins were precipitated by addition of 0.6 g of ammonium sulphate per ml of extract and the mixture was centrifuged at 14,000 rpm for 30 min at 4°C. Proteins were solubilized in 200 µl of 100 mM buffer phosphate (pH 7.0) supplemented with 2 mM β-mercaptoethanol and stored at −80°C. Extracts normally contained around 4 mg ml^−1^ of proteins.

### Detection of Activities of Glycolysis Enzymes and Antioxidant Enzymes

Hexokinase (HK) activity was determined as described in Kerve et al. (2008). HK activity was detected in 1 ml of 50 mM Tris–HCl (pH 8.5) containing 2 mM glucose, 5 mM MgCl_2_, 2.5 mM ATP, 1 mM NAD, 15 mM KCl, 2 U of glucose 6-P dehydrogenase and 10 µg of protein extract. The increase in absorbance at 340 nm, due to synthesis of NADH, was monitored for 30 min. HK activity was determined using the extinction coefficient of NADH (ε = 6.2 mM^−1^ cm^−1^).

Pyruvate kinase (PK) activity was determined as described in Duggleby and Dennis (1973). PK activity was detected in 1 ml of 50 mM HEPES buffer (pH 7.4) containing 50 mM KCl, 10 mM MgCl_2_, 1 mM DTT, 2 mM ADP, 2 mM phosphoenol pyruvate, 0.15 mM NADH, 2 U of lactate dehydrogenase and 10 µg of protein extract. The decrease in absorbance at 340 nm, due NADH oxidation, was monitored for 5 min. PK activity was determined using the extinction coefficient of NADH (ε = 6.2 mM^−1^ cm^−1^).

L-galactono 1,4 lactone dehydrogenase (L-GLDH) activity was detected as described in Mellado et al. (2012). L-GLDH activity was detected 1 ml of 100 mM phosphate buffer (pH 8) containing 3 mM L-galactono 1,4 lactone (Carbosynth, UK), 1 mg ml^−1^ cytochrome c, 0.1 mM KCN, and 50 µg of protein extract. The increase in absorbance at 550 nm, due to cytochrome c reduction, was monitored for 20 min. L-GLDH activity was calculated using the extinction coefficient of reduced cytochrome c (ε = 21 mM^−1^ cm^−1^).

Glutathione synthase (GS) activity was determined as described in Mellado et al. (2012). GS activity was detected in 1 ml of 100 mM phosphate buffer (pH 7.5) containing 150 mM KCl, 20 mM MgCl_2_, 2 mM *γ*-glutamyl-cysteine, 10 mM L-glycine, 5 mM ATP, 2 mM phosphoenol pyruvate, 5 U of pyruvate kinase, 5 U of lactate dehydrogenase, 150 µM NADH and 30 µg of protein extract. The decrease in absorbance at 340 nm, due to NADH oxidation, was monitored for 5 min. GS activity was calculated using the extinction coefficient of NADH (ε = 6.2 mM^−1^ cm^−1^).

Glucose 6-P dehydrogenase (G6PDH) activity was determined as described in González et al. (2010). G6PDH activity was determined in 1 ml of buffer 40 mM Tris–HCl (pH = 8.2) containing 4 mM glucose, 0.6 mM NADP, 5 mM MgCl_2_ and 40 µg of protein extract. The increase in absorbance at 340 nm, due to synthesis of NADPH, was monitored for 5 min. G6PDH activity was calculated using the absorbance coefficient of NADPH (ε = 6.2 mM^−1^ cm^−1^).

### Quantification of ASC and DHA

The alga (100 mg of FT) was frozen with liquid nitrogen and homogenized in a mortar using a pestle. One milliter of 100 mM phosphate buffer (pH 7.0) was added and the homogenization was pursued until thawing. The mixture was centrifuged at 14,000 rpm for 15 min at 4°C and the supernatant was recovered. An aliquot of 10 µl was diluted 10 times and an aliquot of 10 µl of the dilution was mixed with 110 µl of ascorbate assay buffer of the Ascorbic Acid Assay Kit (Abcam, USA), 30 µl of catalysis buffer and 50 µl of the enzyme mix containing 2 µl of the probe. The absorbance was detected at 570 nm using a microplate spectrophotometer (TECAN, USA). To determine the amount of dehydroascorbate, 5 µl of 1 mM DTT were added to an aliquot of 500 µl of the supernatant and incubated at room temperature for 1 h and 5 µl of 5% N-ethylmalimide were added. Absorbance was determined at 570 nm as described previously.

### Quantification of GSH and GSSG

The alga (200 mg of FT) was frozen with liquid nitrogen and homogenized in a mortar using a pestle and 1.2 ml of 0.1% trifluoroacetic acid (TFA)/6.3 mM dietilentriamine pentacetic acid (DTPA) was added. The mixture was centrifuged at 14,000 rpm for 20 min and the supernatant was recovered. The supernatant was filtered through 0.22 µm PVDF membrane and an aliquot of 25 µl of the supernatant was mixed with 45 µl of 200 mM HEPES (pH 8)/6.3 mM DTPA and 1 µl of 25 mM monobromobimane and incubated for 30 min at room temperature in darkness. The reaction was stopped by addition of 30 µl^−1^ mM metasulphonic acid. GSH and GSSG levels were analyzed by High Performance Liquid Chromatography (HPLC) using an Agilent 1260 Infinity and data was compiled using Chemidoc sofware. An aliquot of 20 µl was separated on a reverse phase C-18 column (5 µm particle size, 4.6 mm internal diameter and 15 cm length) at 24°C, eluted using solvent solvent A (0.1% TFA in aqueous solution) and solvent B (100% acetonitile) using a linear gradient (10 min from 0–20%, 30 min from 20–35% and 10 min from 35–100% of solvent B) and a flow rate of 1 ml min^−1^. GSH and GSSG were detected by fluorescence using an excitation wavelength of 380 nm and an emission wavelength of 470 nm. Pure GSH and GSSG were dissolved in filtered water and used as standarts. Retention times of GSH and GSSG were 9.25 and 8.9 min, respectively. The concentrations were calulated using a calibration curve of GSH or GSSG from 0 to 200 nM.

### Quantification of NADPH and NADP

The alga (100 mg of FT) was frozen with liquid nitrogen and homogenized in a mortar using a pestle and 1 ml of lysis buffer at a concentration of 200 mg ml^−1^ from the NAD/NADPH fluorimetric kit (Abcam, USA). The mixture was centrifuged at 14,000 rpm for 10 min and the supernatant was recovered. Two samples of 25 µl of the supernatant were added a 96-well microplate and incubated 15 min at room temperature. The first sample was treated with 25 µl of NADPH extraction solution and incubated 15 min at room temperature and 25 µl of NADP extraction solution were added. The second sample was treated with 25 µl of NADP extraction solution and incubated 15 min at room temperature and 25 µl of NADPH extration solution were added. Both sanples were treated with 75 µl of NADPH reaction mixture and incubated at room temperature for 30 min in darkness. Fluorescence was measured using an excitation wavelengh of 540 nm and and emission wavelength of 590 nm using a microplate spectrophotometer (Tecan, USA). The concentration of NADPH was calculated using a calibration curve of 0–10 µM of NADPH.

### Visualization and Quantification of Superoxide Anions

To visualize superoxide anions, five laminae of the alga were incubated in 2 ml of 100 mM phosphate buffer (pH 7.0) containing 0.1 mM dehydroethydine (DHE) for 40 min in darkness. Algae were washed with 2 ml of filtered seawater to remove DHE excess. 2-hydroxyethidium (2OH-E) was visualized in a confocal microscope (Carl Zeiss model LSM800, Germany) using and excitation wavelenght of 493 nm and emission wavelenght of 590 nm. To quantify superoxide anions, the alga (100 mg of FT) was incubated in 2 mL of phosphate buffer (pH 7) containing 0.1 mM hydroethidine (DHE) for 40 min in darkness. Algae were washed with 2 ml of filtered seawater to remove the excess of DHE. The alga was homogenized in liquid nitrogen using a pestle. One milliter of 40 mM Tris–HCL (pH 7.5) was added and the homogenization was pursued until thawing. The mixture was centifuged at 14,000 for 15 min and the supernatant was recovered. An aliquot of 200 µl was added to a 96-well microplate and the fluorescence was detected using an excitation wavelenght of 480 nm and an emission wavelenght of 590 nm. The concentration was calculated using the extinction coefficient of 2OH-E (ε = 9.4 mM^−1^ cm^−1^).

### Visualization and Quantification of Hydrogen Peroxide

To visualize hydrogen peroxide, five laminae of the alga were incubated in 2 ml of 100 mM phosphate buffer (pH 7.0) containing 0.1 mM 2´,7´-dichlrohydrofluoresceine diacetate (DCHF-DA) for 30 min in darkness. Algae were washed with 2 ml of filtered seawater to remove excess of DCH-DA. Dichlorofluoresceine (DCF) was visualized in a confocal microscope (Carl Zeiss model LSM800, Germany) using and excitation wavelenght of 488 nm and emission wavelenght of 517 nm. To quantify hydrogen peroxide, the alga (100 mg of FT) was incubated in 2 ml of phosphate buffer (pH 7) containing 0.1 mM DCHF-DA for 30 min in darkness. Algae were washed with 2 ml of filtered seawater to remove excess of DCHF-DA. The alga was homogenized in liquid nitrogen using a pestle. One milliliter of 40 mM Tris–HCL (pH 7.5) was added and the homogenization was pursued until thawing. The mixture was centifuged at 14,000 rpm for 15 min and the supernatant was recovered. An aliquot of 200 µl was added to a 96-well microplate and the fluorescence was detected using an excitation wavelenght of 488 nm and an emission wavelenght of 517 nm. The concentration was calculated using a calibration curve of 0–10 µM of DCF.

### Detection of Ion Leakage and Plasma Membrane Damage

The alga (five laminae) were washed with 100 ml milliQ water, quickly transfered to well of a 12-well plate containing 2 ml of 700 mM manitol and incubated for 10 min at 22°C. An aliquot of 50 µl was recovered, conductivity was determined using a compact conductimeter and the aliquot was returned to manitol solution (Horiba Scientific, Japan). The mixture was transferred to 2 ml plastic tubes and treated at 121°C for 20 min and conductivity was measured again in order to determine 100% of conductivity.

### Statistical Analyses

Significant differences were determined by two-way ANOVA followed by Tukey´s multiple comparison tests. Differeneces among mean values were considered to be significant at a probability of 5% (*P <*0.05).

## Results

### Copper-Induced HK Activation and PK Inhibition

**Figure 1:**
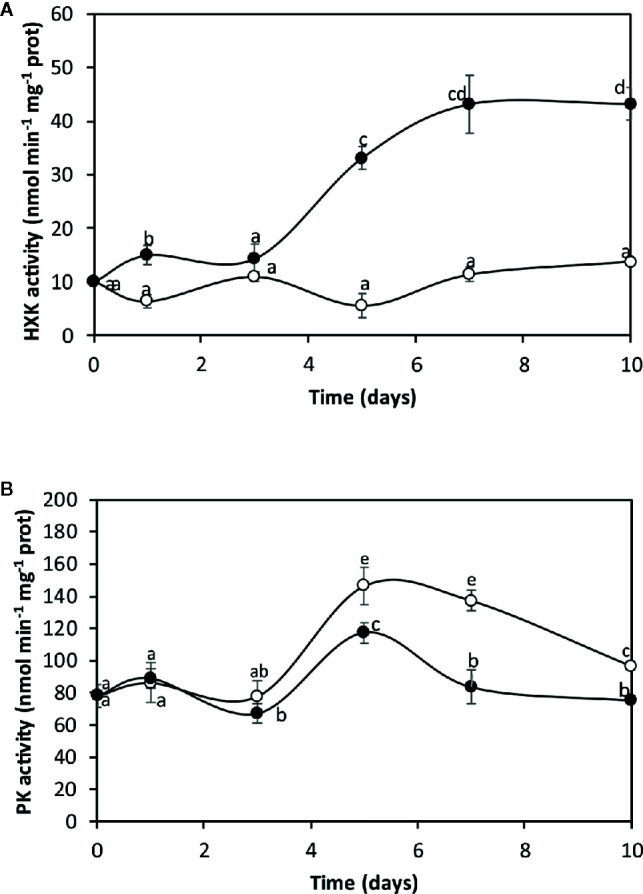
Activities of hexokinase (HK, **A**) and pyruvate kinase (PK, **B**) in the alga cultivated in seawater without copper addition (open circles) and with 10 µM copper (black circles) for 0 to 10 days. Enzyme activities are expressed in nanomoles per minute per milligram of protein extract, and time in days. Symbols represent mean values of three independent experiments. Different letters indicate significant differences among mean values (±SD) (*P <* 0.05).

The alga was cultivated in seawater without copper addition (control) and with 10 µM of copper for 0 to 10 days and the activities of hexokinase (HK), the first enzyme of glycolysis and pyruvate kinase (PK), the last enzyme of glycolysis, were determined (**Figure 1**). HK activity in the alga cultivated in control condition was 10 nmol min^−1^ mg^−1^ of protein at day 0 and it slightly increased to 13.7 nmol min^−1^ mg^−1^ of protein at day 10 (**Figure 1A**). HK activity in the alga cultivated with copper increased from 10 nmol min^−1^ mg^−1^ of protein to 33.1 nmoles min^−1^ mg^−1^ of protein at day 5 and reached a maximal level of 43.3 nmoles min^−1^ mg^−1^ of protein at day 10, which represents an increase of 3.2 times at day 10 (**Figure 1A**).

PK activity in algae cultivated in control condition was 75 nmol min^−1^ mg^−1^ of protein at day 0, it increased to 146 nmol min^−1^ mg^−1^ of protein at day 5 and decreased to 96 nmol min^−1^ mg^−1^ of protein at day 10 (**Figure 1B**). PK activity in the alga cultivated with copper increased from 75 nmol min^−1^ mg^−1^ of protein to 117 nmol min^−1^ mg^−1^ of protein at day 5 and decreased to 75 nmol min^−1^ mg^−1^ of protein at day 10, which represent a inhibition of 28% at day 10 (**Figure 1B**). Thus, the increase in HK and the inhibition of PK indicate that glycolysis is inhibited at its final step that may lead to carbon flux reprogramming.

### Copper-Induced L-GLDH and G6PDH Activation

**Figure 2:**
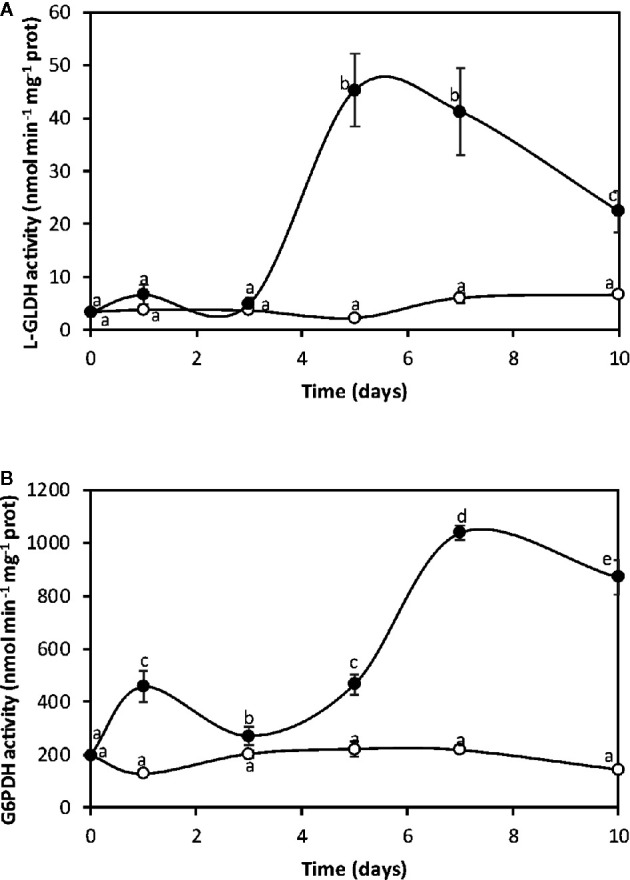
Activities of L-galactone 1,4 lactone dehydrogenase (**A**, L-GLDH) and glucose 6-P dehydrogenase (**B**, G6PDH) in the alga cultivated in seawater without copper addition (open circles) and with 10 µM copper (black circles) for 0 to 10 days. Enzyme activities are expressed in nanomoles per minute per milligram of protein extract, and time in days. Symbols represent mean values of three independent experiments. Different letters indicate significant differences among mean values (±SD) (*P <* 0.05).

Considering that glycolysis is inhibited in its final step, it is posible that carbon flux may be redirected to increase L-GLDH, that synthesize ASC from glucose 6-P, and G6PDH, that uses glucose 6-P as substrated and synthesize NADPH. The activity of L-GLDH in control algae was 2 nmol min^−1^ mg^−1^ of protein at day 0 and it increased to 6.6 nmol min^−1^ mg^−1^ of protein at day 10 (**Figure 2A**). L-GLDH activity in the alga cultivated with copper increased from 2 nmol min^−1^ mg^−1^ of protein to 45 nmol min^−1^ mg^−1^ of protein at day 5 and decreased to 22 nmol min^−1^ mg^−1^ of protein at day 10, which represents an increase of 3.3 times at day 10 (**Figure 2A**). The activity of G6PDH in control algae was 200 nmol min^−1^ mg^−1^ of protein at day 0 and it decreased to 143 at day 10 (**Figure 2B**). G6PDH activity in the alga cultivated with copper increased from 200 nmol min^−1^ mg^−1^ of protein at day 0 to 440 nmol min^−1^ mg^−1^ of protein at day 1, increased again at day 5 and reached a maximal level of 1,039 nmol min^−1^ mg^−1^ of protein at day 7 and then decreased to 871 nmol min^−1^ mg^−1^ of protein at day 10, which represents an increase of 6.1 times at day 10 (**Figure 2B**). Thus, there is a carbon flux reprogramming since G6-P is used to synthesize ASC, and NADPH due to G6PDH activation.

### Copper-Induced HK Activation and PK Inhibition Is Dependent on Activation of CDPKs and MAPKs

**Figure 3:**
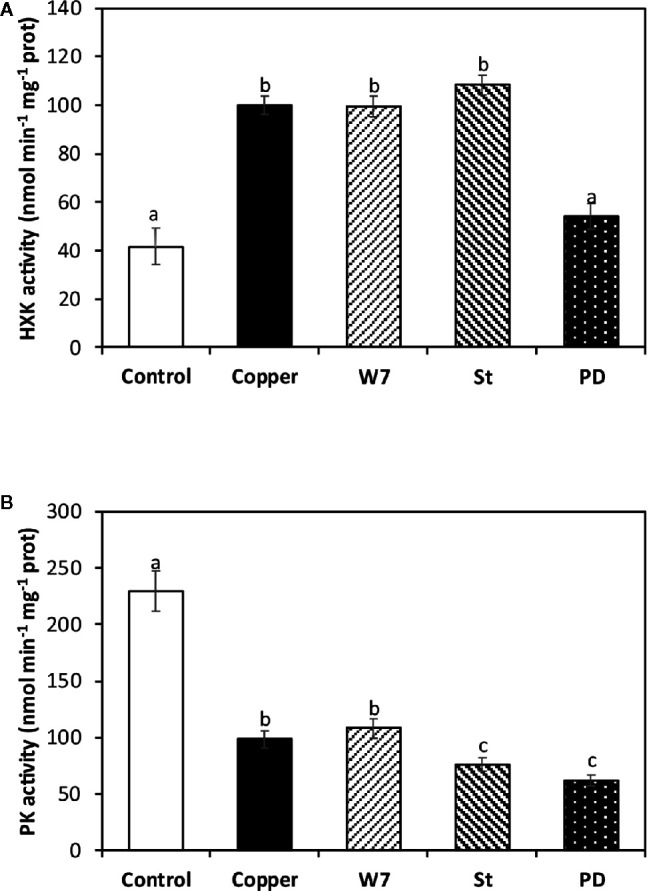
Activities of enzymes hexokinase (HK, **A**) and pyruvate kinase (PK, **B**) cultivated in seawater without copper addition for 5 days (open bars) and with 10 µM of copper and with 100 µM of W-7 and 10 µM copper, 10 µM staurosporine (St) and 10 µM copper, and with 5 µM of PD-98059 (PD) and 10 µM copper for 5 days. Enzyme activities are expressed in nanomoles per minute per milligram of protein extract. Bars represent mean values of three independent experimets. Different letters indicate significant diferences among mean values (±SD) (*P <* 0.05).

The alga was cultivated in seawater without copper addition (control), with 10 µM of copper, and with an inhibitor of CaMKs, W-7, an inhibitor of CDPKs, staurosporine (St), and an inhbitor of MAPKs, PD-98059 (PD), and with 10 µM of copper for 5 d. HK activity in the alga cultivated in control condition was 100 nmol min^−1^ mg^−1^ of protein at day 5 (**Figure 3A**). HK activity in the alga treated with W-7 and (St) and copper was not significantly different from HK activity in control alga. In contrast, HK activity in the alga treated with PD and copper was 54 nmol min^−1^ mg^−1^ of protein, which is significantly different and represent an inhibition of 46% (**Figure 3A**). Thus, HK activation is dependent on activation of MAPKs.

The activity of PK in the alga cultivated in control condition was 229 nmol min^−1^ mg^−1^ of protein and in the alga treated only with copper it was 98 nmol min^−1^ mg^−1^ of protein (**Figure 3B**). PK activity in the alga treated with W-7 and copper was not significantly different to the activity found in the alga treated only with copper. In contrast, PK activity was 75 nmol min^−1^ mg^−1^ of protein in the alga treated with W-7 and copper, and it was 62 nmol min^−1^ mg^−1^ of protein and in the alga treated with PD and copper, which represents an inhibition of 23.4 and 36.7%, respectively (**Figure 3B**). Thus, the inhibition of PK activity is dependent on the activation of CDPKs and MAPKs.

### Copper-Induced L-GLDH, GS and G6PDH Activation Is Dependent on Activation of CDPKs and MAPKs

**Figure 4:**
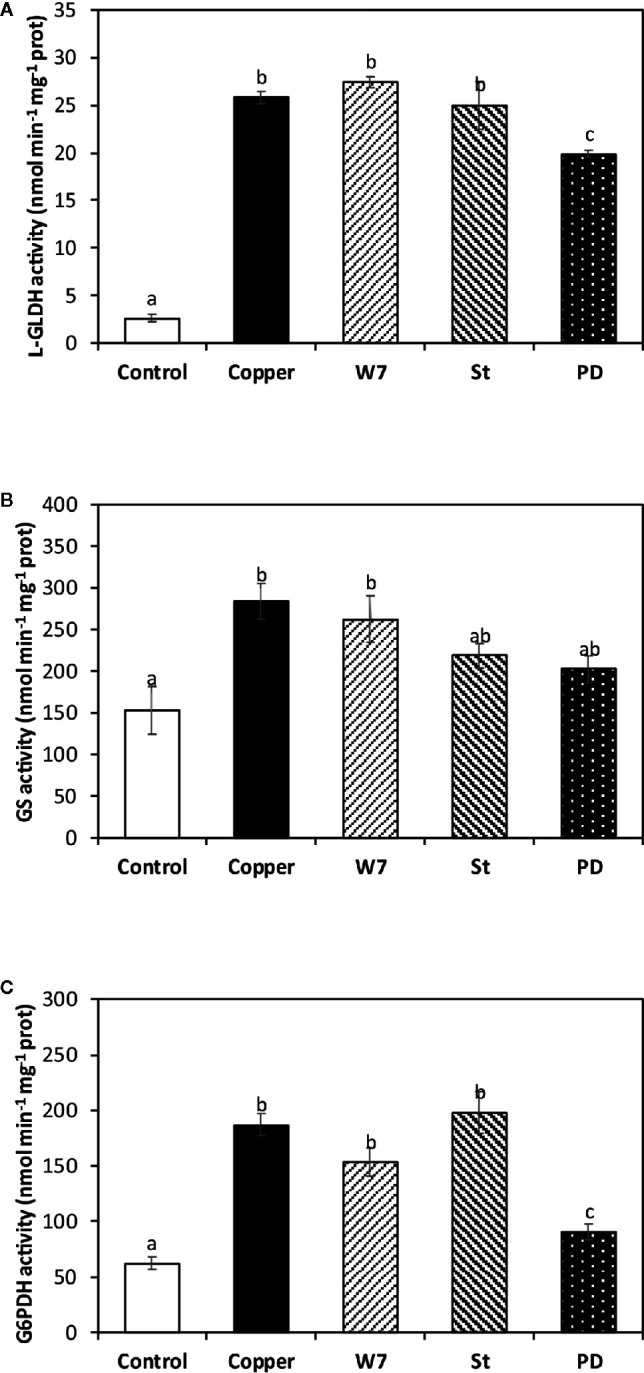
Activities L-galactono 1,4 lactone dehydrogenase (L-GLDH, **A**), glutathione synthase (GS, **B**) and glucose 6-P dehydrogenase (G6PDH, **C**) cultivated in seawater without copper addition for 5 days (open bars), with 10 µM of copper and with 100 µM of W-7, 10 µM staurosporine (St), 5 µM of PD-98059 (PD) and 10 µM copper for 5 days (hashed bars). Enzyme activities are expressed in nanomoles per minute per milligram of protein extract. Bars represent mean values of three independent experimets. Different letters indicate significant diferences among mean values (±SD) (*P <* 0.05).

The alga was cultivated in seawater without copper addition (control), with 10 µM of copper, and with inhibitors of CaMKs, CDPKs and MAPKs pathways and with 10 µM of copper for 5 d, and the activities of L-GLDH, GS and G6PDH were determined. L-GLDH activity in control alga was 2.6 nmol min^−1^ mg^−1^ of protein and in the alga treated only with copper and it was 186 nmol min^−1^ mg^−1^ of protein at day 5 (**Figure 4A**). L-GLDH activity was not significantly different to the activity in the alga treated with W-7 or St and with copper. In contrast, L-GLDH activity was 19.9 nmol min^−1^ mg^−1^ of protein in the alga treated with PD and copper, which represents an inhibition of 23%, at day 5 (**Figure 4A**). Thus, the increase in L-GLDH activity is due to activation of MAPKs.

GS activity in control alga was 152 nmol min^−1^ mg^−1^ of protein and it was 284 nmol min^−1^ mg^−1^ of protein in the alga treated only with copper (**Figure 4B**). GS activity was not significantly different to the activity in the alga treated with W-7 and copper whereas it was 218 nmol min^−1^ mg^−1^ of protein in the alga treated with St and copper and it was 202 nmol min^−1^ mg^−1^ of protein in the alga treated with PD and copper, which represent an inhibition of 23 and 29%, respectively, at day 5 (**Figure 4B**). Thus, the increase in GS activity is dependent on the activation of CDPKs and MAPKs.

G6PDH activity in the alga cultivated in control condition was 62 nmol min^−1^ mg^−1^ of protein and in the alga treated only with copper it was 186 nmol min^−1^ mg^−1^ of protein (**Figure 4C**). G6PDH activity was not significantly different in the alga treated with W-7 or St and with copper, whereas it was 90 nmol min^−1^ mg^−1^ of protein in algae treated with PD and copper, which represents an inhibition 51.6%, at day 5 (**Figure 4C**). Thus, the increase in G6PDH activity is dependent on the activation of MAPKs.

### Copper-Induced Increase in the Level of ASC and GSH, but not NADPH, is Dependent on the Activation of CaMK, CDPKs or MAPKs

**Figure 5:**
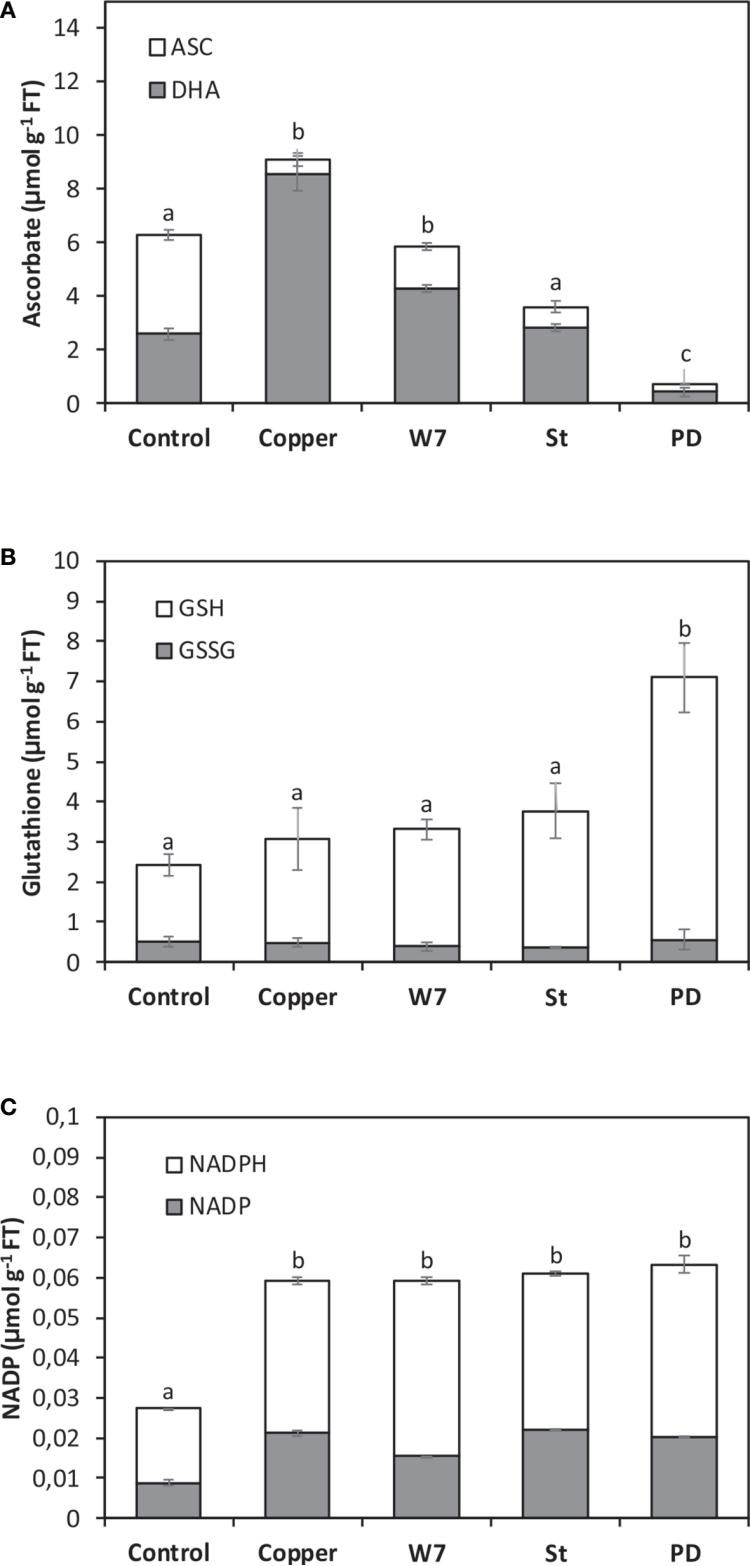
Level of total ascorbate (ASC, **A**), total glutathione (GSH, **B**) and total NADPH (**C**) corresponding to ASC, GSH and NADPH levels (white bars) and dehydroascorbate (DHA), oxidized glutathione (GSSG) and NADP (black bars) in *U. compressa* cultivated in seawater without copper addition for 5 days, with 10 µM of copper for 5 days, and with 100 µM of W-7, 10 µM staurosporine (St), with 5 µM of PD-98059 (PD) and 10 µM copper for 5 days (hashed bars). Levels of ASC, DHA, GSH, GSSG, NADPH and NADP are expressed in micromoles per gram of fresh tissue (FT). Bar represent mean values of three independent experiments. Different letters indicate significant differences among mean values (±SD) (*P <* 0.05).

The alga was cultivated in seawater without copper addition (control), with 10 µM of copper, with inhibitors of CaMKs, CDPKs and MAPKs and with 10 µM of copper, for 5 d and the levels of ASC/DHA, GSH/GSSG and NADPH/NADP were determined. The levels of ASC and DHA in control alga were 4 and 2.2 µmoles g^−1^ of FT, respectively, and in the alga treated only with copper these levels were 0.6 and 8.5 µmoles g^−1^ of FT, respectively, at day 5. The level of total ASC (ASC + DHA) in control algae was 6.2 µmoles g^−1^ of FT and in the alga treated only with copper it was 9.1 µmoles g^−1^ of FT (**Figure 5A**). Total ASC significantly decreased in the alga treated with W-7, St or PD and with copper to 5.8, 4,4 and 0.7 µmoles g^−1^ of FT which represents a decrease of 56, 41 and 88%, respectively (**Figure 5A**). Thus, total ASC level is dependent on the activation of CaMKs, CDPKs and MAPKs.

The levels of GSH and GSSG in control alga were 1.9 and 0.5 µmoles g^−1^ of FT, respectively, and in the alga treated only with copper these levels were 2.5 and 0.5 µmoles g^−1^ of FT, respectively, at day 5 (**Figure 3B**). The level of total GSH (GSH + GSSG) in control alga was 2.4 µmoles g^−1^ of FT and in the alga treated only with copper it was 3 µmoles g^−1^ of FT (**Figure 5B**). The levels of total GSH in algae treated with W-7 or St and with copper were 3.3 and 3.7 µmoles g^−1^ of FT, respectively, but these levels were not significantly different compare to the level in the alga treated only with copper (**Figure 5B**). The level of total GSH in algae treated with PD and copper was 7.1 µmoles g^−1^ of FT and this level was significantly higher than the level in algae treated only with copper (**Figure 5B**). Thus, the level of total GSH is dependent on activation of MAPKs.

The levels of NADPH and NADP in control alga were 8 and 19 nmoles g^−1^ of FT, respectively, and in the alga treated only with copper these levels were 21 and 38 nmoles g^−1^ of FT, respectively, at day 5 (**Figure 5C**). The level of total NADPH (NADPH + NADP) in control algae was 26 nmoles g^−1^ of FT and in algae treated with copper it was 59 nmoles g^−1^ of FT (**Figure 5C**). The levels of total NADPH in algae treated with W-7, St or PD and with copper were 59, 60 and 63 nmoles g^−1^ of FT, respectively, but they were not significantly different compare to the level in the alga treated only with copper. Thus, the level of total NADPH is not dependent on the activation of CaMKs, CDPKs or MAPKs.

### Copper-Induced Increased Levels of Superoxide Anions and Hydrogen Peroxide Are Dependent on Activation of CaMKs, CDPKs and MAPKs

**Figure 6:**
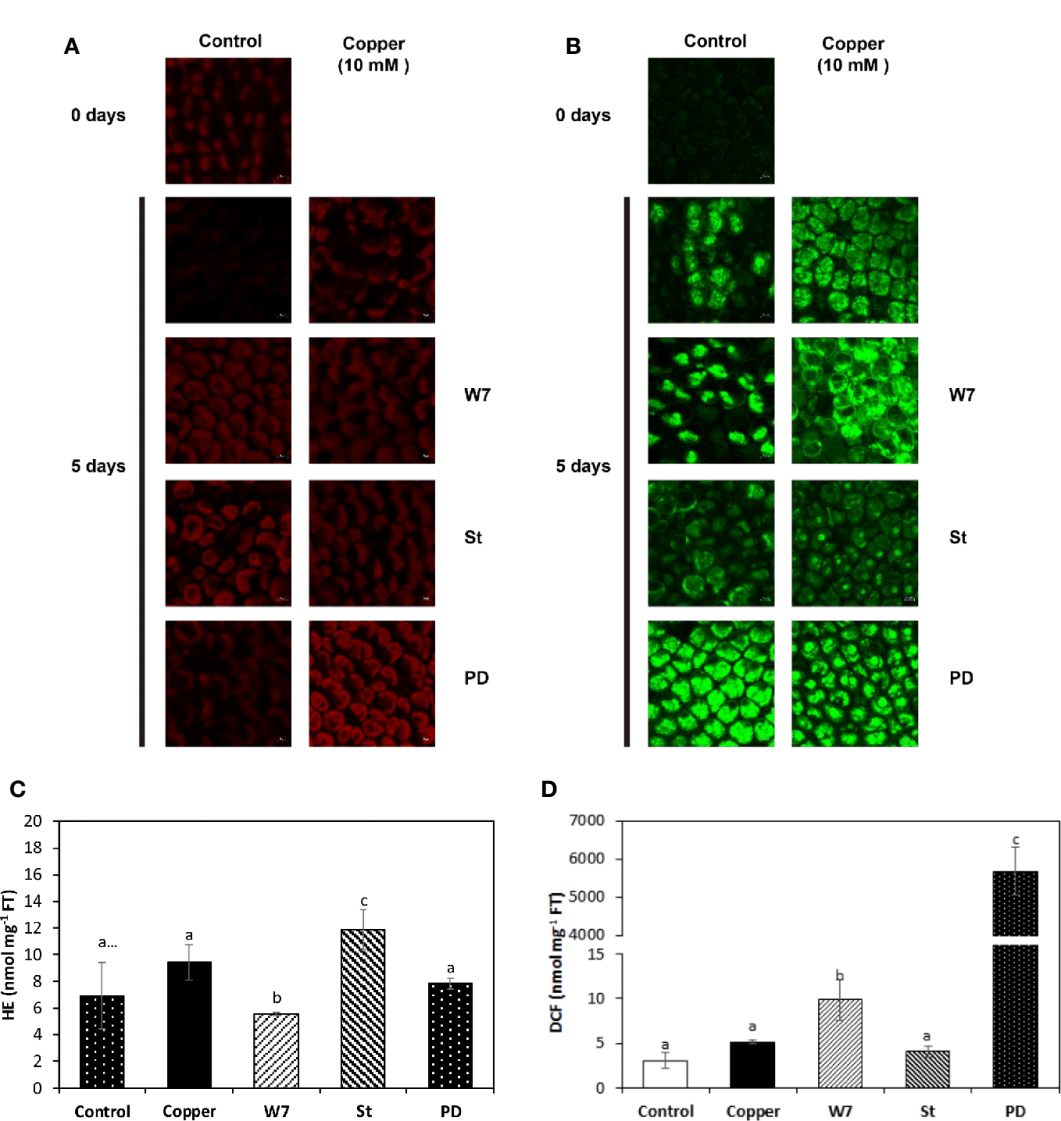
Visualization of superoxide anions (SA, **A**) and hydrogen peroxide (HP, **B**) by confocal microscopy in *U. compressa* cultivated in seawater without copper addition (control), with 10 µM copper (copper), with 100 µM of W-7, 10 µM staurosporine (St), 5 µM PD-98059 (PD) and copper for 5 days. Levels of SA **(C)** and HP **(D)** in the alga cultivated in seawater without copper addition for 5 days (white bars), with 10 µM of copper (black bar), with 100 µM W-7, 10 µM St, 5 µM PD and 10 µM of copper for 5 days (hashed bars). Level of superoxide anions and hydrogen peroxide are expressed in nanomoles per milligram of fresh tissue (FT). Bars indicate mean values of three independent experiments. Different letters indicate significant differences among mean values (±SD) (*P <* 0.05).

The level of superoxide anion (SA) in control alga was 6 nmoles g^−1^ of FT and it was 9 nmoles g^−1^ of FT in the alga treated with copper at day 5, but these levels were not significantly different among them and the fluorescence was observed mainly in the chloroplast (**Figures 6A, C** **)**. The level of SA in algae treated with W-7 and copper was 5 nmoles g^−1^ of FT, which is lower than the level in the alga treated only with copper and the fluorescence is located mainly in the chloroplast of the cells (**Figures 6A, C** **)**. The level of SA in the alga treated with St and copper was 12 nmoles g^−1^ of FT which is higher than the level in the algae treated with copper and fluorescence is located mainly in the chloroplast (**Figures 6A, C** **)**. The level of SA in the alga treated with PD and copper was 8 nmoles g^−1^ of FT, this level is not significantly different from the level in the alga treated only with copper and the fluorescence is located mainly in the chloroplast. (**Figures 6A, C** **)** Thus, the level of SA is not different in control and copper-treated algae indicating that oxidative stress is efficiently buffered and that the increase in SA level is dependent on activation of CaMK and CDPKs.

The level of hydrogen peroxide (HP) in algae cultivated in control condition was 3 nmoles g^−1^ of FT and it was 5 nmoles g^−1^ of FT in the algae treated only with copper at day 5, but these levels were not significantly different and the fluorescence was located in the whole cell (**Figures 6B, D** **)**. The level of HP in the alga treated with W-7 and copper was 9 nmoles g^−1^ of FT and this level was significantly higher than the level in the alga treated only with copper and the fluorescence was located mainly in the chloroplast (**Figures 6B, D** **)**. The level of HP in the alga treated with St and copper was 4 nmoles g^−1^ of FT and this level was not significantly higher than the level in the alga treated only with copper but the fluorescence was located mainly in the nucleus (**Figures 6B, D** **)**. The level of HP in the alga treated with PD and copper was 5,700 nmoles g^−1^ of FT and this level was significantly higher than the level in the alga treated only with copper and the fluorescence was located mainly in the chloroplast and the nucleus (**Figures 6B, D** **)**. Thus, the level of HP is efficiently buffered in algae treated only with copper and the increase in HP level is dependent on the activation of CaMKs and more significantly on the activation of MAPKs.

### Copper-Induced Protection of Plasma Membrane Integrity Is Dependent on the Activation CaMKs, CDPKs and MAPKs

**Figure 7:**
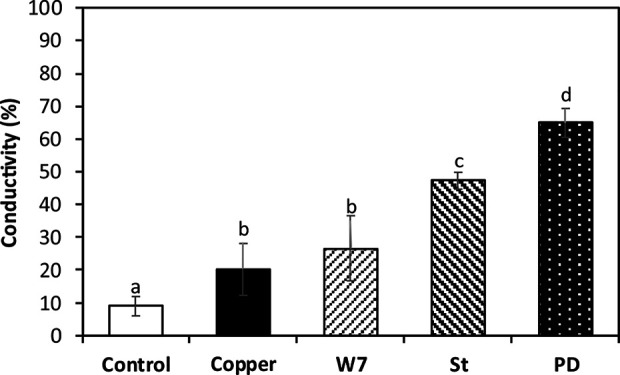
Percentage of ion leakage in *U. compressa* cultivated in seawater without copper addition (white bar), with 10 µM of copper (black bar), with 100 µM of W-7, 10 µM of staurosporine (St), 5 µM of PD-98059 (PD) and with copper for 5 days (hashed bars). Percentage of ion leakage is obtained considering 100% ion leakage in obtained in the alga subjected to heating at 120°C for 20 min. Bar represent mean values of three independent experiments. Different letters indicate significant differences among mean values (±SD) (*P <* 0.05).

The level of ion leakage (IL) in control algae at day 5 was 8.9% compare with algae subjected to 120°C for 20 min (100%). IL was 20.3% in the alga treated only with copper which is significantly higher than the level in control alga (**Figure 7**). IL in the alga treated with W-7 and copper was 26.6%, in the alga treated with St and copper it was 47.3% and in the alga treated with PD and copper it was 65% which and these percentages were significantly different compare to the percentage in the alga treated only with copper (**Figure 7**). Thus, plasma membrane integrity in the alga treated with copper is dependent on the activation of CaMKs, CDPKs and MAPKs.

## Discussion

### Copper-Induced Increase in HK, L-GLDH, GS and G6PDH Activities and Decrease in PK Activity Is Dependent on CDPKs and MAPKs

In this work, we initially showed that copper induces the increase in HK activity through MAPKs activation whereas it inhibits PK through CDPKs and MAPKs activation indicating that phosphorylation of HK and PK enzymes regulate these activities. In this sense, it has been shown that PK activity is inhibited by phosphorylation of ser215 in soybean and cotton cells and the additional phosphorylation in ser402 enhances its degradation in the proteasome (Tang et al., 2003; Zhang and Liu, 2017). In addition, it has been shown that HK1 and HK2 are activated by c-Src tyrosine kinase that phosphorylates tyr73, which favors proliferation, migration and invasion of human tumoral cells (Zhang et al., 2017). Thus, HK and PK activities are regulated by phosphorylation in the marine alga *U. compressa*, as it has been shown in plants and animals. In addition, the increase in HK activity and the decrease in PK activity may enhance the level of G6-P that is produced by HK and the level of 3-phosphoglycerate that is produced by phospho-glycerate kinase, an enzyme located before PK in glycolysis. In addition, the increase in G6-P may enhance the synthesis of ASC and the activity of G6PDH since glucose 6-P is the precursor of ASC and the substrate of G6PDH that produces NADPH. In addition, the increase in 3-phosphoglycerate may enhance the synthesis of amino acids such as cysteine and glycine that are required for GSH synthesis. Moreover, the amino acid glutamate is synthesized from 2-oxoglutarate that is produced by the enzyme isocitrate dehydrogenase (IDH) in the Krebs cycle. In this sense, it was determined that IDH activity was increased in response to copper excess (data not shown). The increase in IDH activity may enhance the level of 2-oxoglutarate that is required for glutamate synthesis which, in turn, is used for the synthesis of NADPH and GSH. Furthermore, the activity of G6PH and GS were also increased in response to copper stress (this work). Thus, the increase in glutamate, cysteine and glycine levels as well as in G6PDH and GS activities may explain the increase NADPH and GSH level. Thus, copper-induced HK activation and PK inhibition lead to carbon flux reprogramming resulting in an increase of ASC, GSH and NADPH levels.

### Copper-Induced Increase in the Level of ASC and GSH, but Not NADPH, Is Dependent on the Activation of CaMK, CDPKs or MAPKs

**Figure 8:**
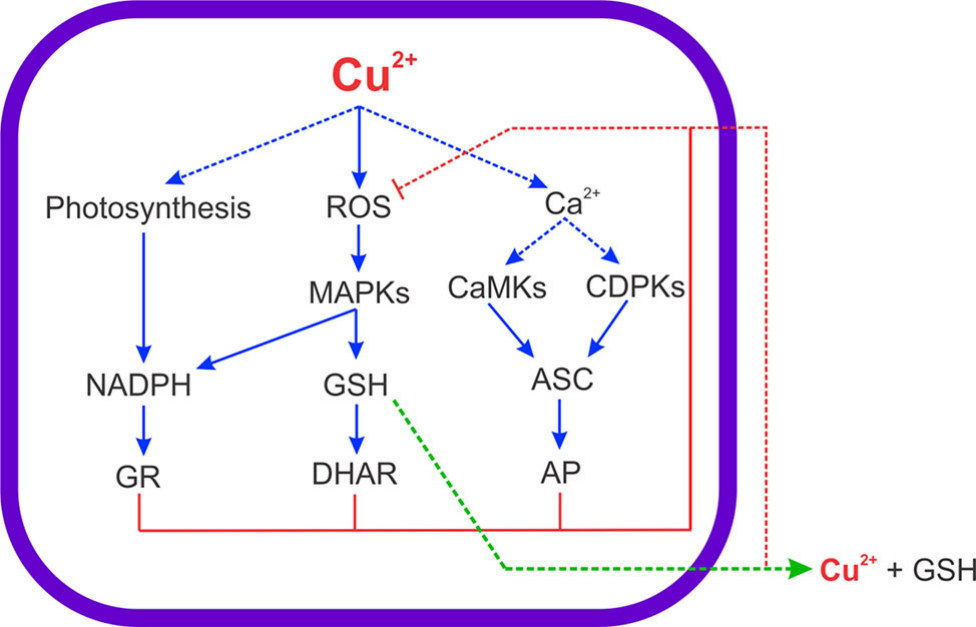
Scheme of copper-induced increase in ROS and intracellular calcium levels leading to activation of MAPK, CDPKs and CaMKs signaling pathways, which coupled to the increase in net photosynthesis, allow the increase in ASC, GSH and NADPH levels to cope with copper-induced oxidative stress in the marine alga *U. compressa*. Arrows in blue indicate activation, arrows in red indicate inhibition, the arrow in green indicates extrusion, and choppy arrows indicate results that do not belong to this study.

Here, it was shown that ASC synthesis is dependent on CaMKs, CDPKs and MAPKs activation (see scheme in **Figure 8**) indicating that ASC synthesis is regulated by phosphorylation. Until now, it is not known how activation of CaMKs, MAPKs or CDPKs pathways can stimulate ASC synthesis in plants or algae. However, a direct phosphorylation of an enzyme involved in ASC synthesis may occur and/or the phosphorylation of transcription factor involved in the expression of an enzyme that participates in ASC synthesis, but the latter need to be further investigated. ASC is also the substrate of AP and this activity was strongly increased in the alga treated with copper (Mellado et al., 2012). Even if AP activity is increased, the level of ASC remained enhanced in the alga exposed to copper excess indicating that there is a strong synthesis of ASC in *U. compressa*. On the other hand, the level of GSH was dependent on activation of MAPKs. In this sense, it has been shown that inhibition of ERK, JNK or p38 MAPKs induced a decrease in the level of GSH in *U. compressa* exposed to copper excess (Rodríguez-Rojas et al., 2019). Thus, the activation of the three MAPK pathways are involved in the increase of GSH synthesis. In addition, GSH is the substrate of DHAR and this activity may also be increased in *U. compressa* since AP and GR are increased (González et al., 2010; Mellado et al., 2012). In addition, it has been shown that copper is released to the extracellular medium with GSH in equimolar amounts (Navarrete et al., 2019). Thus, GSH may be also required to extrude copper from *U. compressa* as well as to inhibit copper-induced ROS accumulation and oxidative stress (see scheme in **Figure 8**).

Surprisingly, the level of NADPH is not dependent on activation of CaMK, CDPKs and MAPKs. Considering that G6PDH is regulated by CaMKs and MAPKs and that the level of NADPH did not change in the alga treated with inhibitors and copper, it is possible that NADPH produced by G6PDH is used GR activity in the cytoplasm (see scheme in **Figure 8**). In addition, NADPH produced by G6PDH may be consumed to increase C, N and S assimilation, which are processes requiring NADPH, in the cytoplasm (Kopriva et al., 1999; Kopriva et al., 2002). In this sense, it has been shown that C, N and S assimilation is increased in the alga exposed to copper excess since the level of transcripts and the activities of key enzymes involved in these processes are also increased (Laporte et al., 2020). The enhanced C, N and S assimilation may be required to the synthesis of new proteins and membranes that are damaged by copper-induced oxidative stress. In addition, the constant level of NADPH that did not change with inhibitors of CaMKs, CDPKs and MAPKs indicates that the constant NADPH level is due to photosynthesis. In this sense, it was shown that net photosynthesis is increased in the alga exposed to copper excess for 0 to 5 days (Laporte et al., 2020) and the increase in NADPH level is due to activation of ferredoxin-NADPH reductase (FNR) in chloroplasts. It has been shown FNR is not regulated by phophorylation but instead by acetylation of a lysine residue located near the active site in *A. thaliana* (Lethimäki et al., 2014). The latter may explain the absence of regulation of FNR by phosphorylation due to CaMK, CDPKs or MAPKs activation.

### Copper-Induced Levels of ROS and Membrane Damage Are Dependent on Activation of CaMKs, CDPKs and/or MAPKs

On the other hand, the level of SA and HP were not increased in the alga exposed only to copper excess (this work). The latter indicates that efficient antioxidant mechanisms were induced in the alga in response to copper that are able to cope with copper-induced oxidative stress. In addition, inhibitors of CaMKs, CDPKs, MAPKs lead to increased levels of SA and HP and to the damage of cellular membranes. Thus, the accumulation of SA and HP are the main responsible of cellular membrane damage observed in the alga treated with copper (González et al., 2010; González et al., 2012). It is interesting to mention that HP is located in the whole cell in alga treated only with copper whereas HP is located in the nucleus, and not the chloroplast or the cytoplasm, in the alga treated with CDPKs inhibitor and copper. The latter may indicate that CDPKs are involved in the inhibition of antioxidant enzymes in the chloroplast and the cytoplasm. In addition, the inhibition of MAPKs strongly increased the level of HP in the chloroplast and the nucleus suggesting that MAPKs are involved in the activation of antioxidant enzymes in the chloroplast and the nucleus. The latter is in accord with previous findings showing that the levels of transcripts encoding antioxidant enzymes such as SOD, CAT and AP are strongly decreased by inhibitors of ERK, JNK and p-38 MAPKs indicating that the decrease in activities of antioxidant enzymes is due to inhibition of gene expression (Rodríguez-Rojas et al., 2019). Thus, the mitigation of copper-induced oxidative stress is due mainly to the activation of antioxidant enzymes and the synthesis of ASC, GSH and NADPH. However, concentrations higher than 50 µM of copper can surpassed the mechanisms for buffering copper-induced oxidative stress leading to cell death in *U. compressa* (González et al., 2010). Thus, copper-induced oxidative stress is buffered by the increase in activities of antioxidant enzymes and in the synthesis of ASC, GSH and NADPH and the latter is dependent on the activation of CaMK, CDPKs and MAPKs signaling pathways in *U. compressa*.

## Conclusion

Copper induced the activation of MAPKs, CDPKs and CaMKs leading to the modulation of glycolysis and induction of carbon flux reprogramming which trigger an increase in ASC, GSH and NADPH syntheses and the activation of antioxidant enzymes in order to buffer copper-induced oxidative stress and to inhibit cell membrane damage.

## Data Availability Statement

The datasets presented in this study can be found in online repositories. The names of the repository/repositories and accession number(s) can be found below: 10.6084/m9.figshare.12142101.

## Author Contributions

DL did experimental work. AG helped with results analyses. AM helped with experimental design and wrote the manuscript.

## Funding

This work was financed by Postdoctoral Fondecyt 3170511 to DL and by VRIDEI-USACH.

## Conflict of Interest

The authors declare that the research was conducted in the absence of any commercial or financial relationships that could be construed as a potential conflict of interest.
